# Distal arch replacement for aortic aneurysm associated with pseudocoarctation through the L-incision approach

**DOI:** 10.1093/icvts/ivac094

**Published:** 2022-04-13

**Authors:** Takuya Goto, Junichi Koizumi, Hirofumi Saiki, Hajime Kin

**Affiliations:** 1 Department of Cardiovascular Surgery, Iwate Medical University, Morioka, Japan; 2 Department of Pediatrics, Iwate Medical University, Morioka, Japan

**Keywords:** Pseudocoarctation, Aortic aneurysm, Descending aortic perfusion, L-incision approach

## Abstract

We report the case of a 16-year-old boy in whom we successfully repaired a distal aortic arch aneurysm associated with pseudocoarctation using double aortic cannulation and antegrade selective cerebral perfusion through the L-incision approach. This approach provided excellent exposure from the ascending aorta to the descending aorta, which enabled total body perfusion. We avoided cardiac arrest and hypothermic circulatory arrest during the surgery. The L-incision approach could be a better alternative for aortic arch surgery in adolescents.

## INTRODUCTION

Pseudocoarctation of the aorta is a rare congenital anomaly characterized by kinking and buckling of the aorta without stenosis. There have been some reports [1, 2] of aneurysmal rupture and various surgeries. Several surgical approaches are indicated based on the procedure performed, such as median sternotomy [2] and left thoracotomy [3], on a case-by-case basis. Here, we report a case of successfully repaired distal arch aneurysm associated with pseudocoarctation of the aorta in an adolescent using double aortic cannulation and antegrade selective cerebral perfusion through the L-incision approach. To the best of our knowledge, this approach has never been described for the management of pseudocoarctation of the aorta.

## CASE

**Figure 1:**
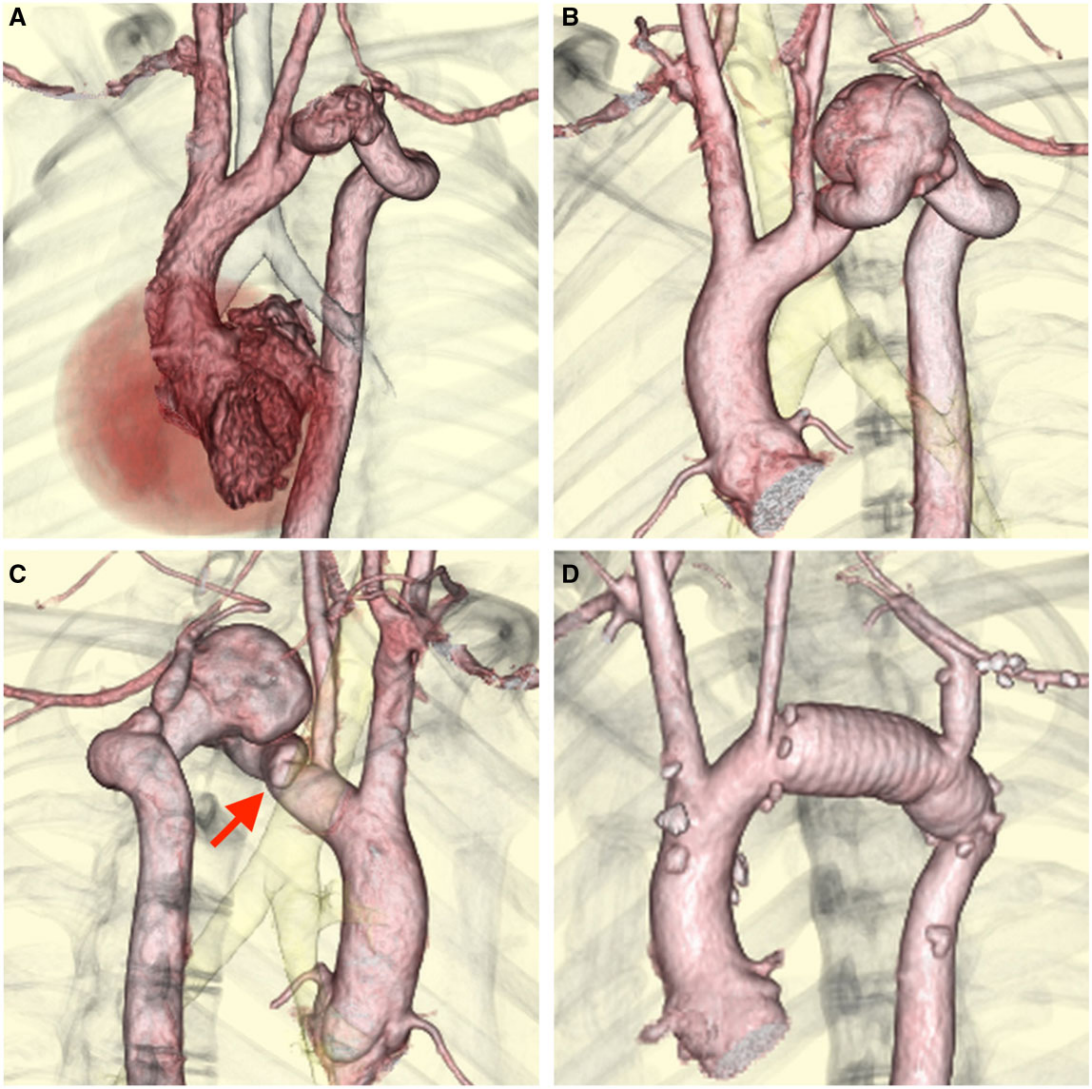
(**A**) Computed Tomography (CT) angiography at 8 years of age revealed pseudocoarctation of the aorta without aneurysm formation or significant stenosis. (**B** and **C**) Preoperative CT angiography revealed saccular aneurysm of the distal aortic arch. Kinking and a tortuous aorta is observed just beneath the left common carotid artery (arrow). (**D**) Postoperative CT angiography revealed a well-replaced distal aortic arch and reconstructed left subclavian artery.

A 16-year-old boy with a distal arch aneurysm associated with pseudocoarctation of the aorta was admitted to our hospital for elective surgery. He had been referred to our hospital at 8 years of age. Chest computed tomography at referral had revealed a kinked, elongated, tortuous distal aortic arch without aneurysm formation or significant stenosis (Fig. 1A). The diameter of the distal aortic aneurysm increased to 34 mm during the 8-year observation, and kinking and a tortuous aorta were identified just beneath the left common carotid artery (LCCA) (Fig. 1B and C); therefore, elective surgery was scheduled through a multidisciplinary team discussion.

**Figure 2:**
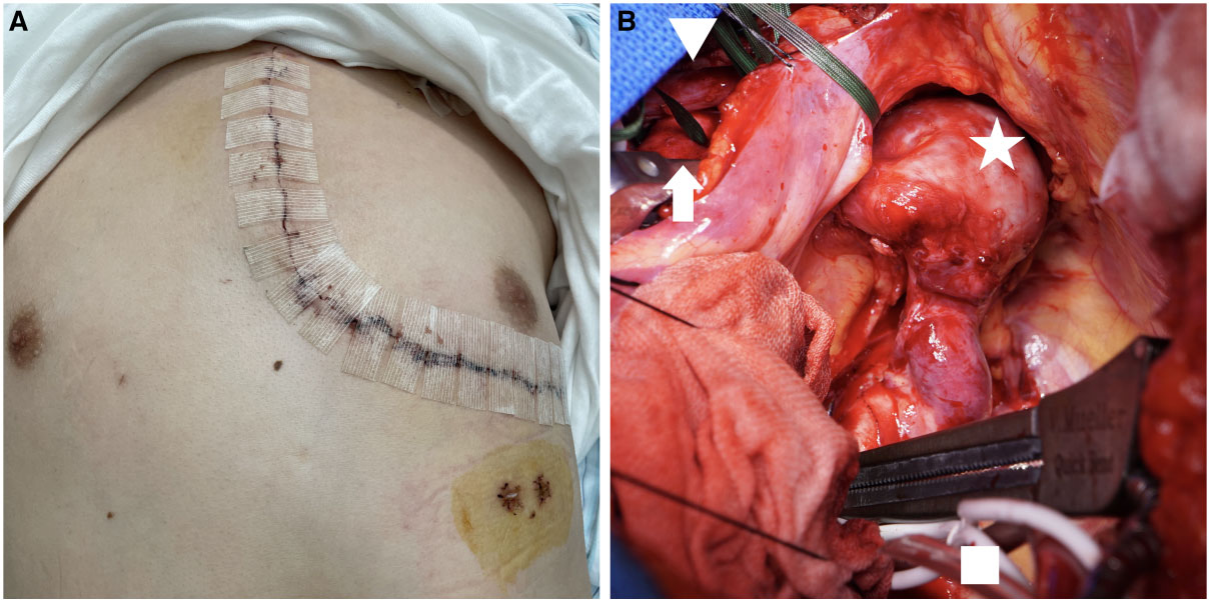
(**A**) Skin incision in the L-incision approach. (**B**) Intraoperative photograph: arrow, innominate artery; arrow head, innominate vein; star, aortic aneurysm; square, cannula in the descending aorta.

During surgery, the L-incision approach (Fig. 2A) (4th intercostal left anterior thoracotomy and upper half-median sternotomy) was performed for good visualization of the ascending and descending thoracic aorta. A spring retractor was used to retract the left upper sternum. Full-flow cardiopulmonary bypass was established with double ascending and descending aortic perfusion and single right atrial drainage to facilitate the emptying of the heart and better exposure of the descending aorta. The clamps were applied to the aortic arch between the innominate artery and LCCA, and the distal descending aorta and left subclavian artery (LSCA). The aneurysmal wall appeared extremely thinned (Fig. 2B). We transected aortic arch just distal of the LCCA and introduced the perfusion cannula to the LCCA from opened aortic incision for selective cerebral perfusion (250 ml/min). Replacement of the distal arch aneurysm with LSCA reconstruction was performed with a one-branched knitted polyester graft (Triplex 20/9 mm, Terumo Corporation, Tokyo, Japan) under a beating heart without circulatory arrest (Video 1). No blood transfusions were required. The pathological findings of the resected aneurysm revealed destruction of the medial elastic fibre without cystic medial necrosis.

The postoperative course was uneventful, with 16-h ventilator support and 2-day intensive care unit stay. Postoperative computed tomography revealed excellent anastomosis with neither residual stenosis nor aneurysmal lesion (Fig. 1D). The patient was discharged on the 8th postoperative day with no neurological complications.

## DISCUSSION

The L-incision approach was described by Tominaga *et al.* [4] for total aortic arch replacement. They emphasized that improved visualization of the distal descending aorta and LSCA facilitated the better performance of the anastomosis. Several advantages of the L-incision approach have been proposed for a patient with pseudocoarctation. First, good visualization of both the ascending and descending aorta enables direct double aortic cannulation to avoid circulatory arrest or peripheral artery cannulation, which has the risk of lower body or leg ischaemia, especially in young patients with small peripheral arteries. Second, LSCA, which is sometimes dislocated in a patient with pseudocoarctation, could be reconstructed with better exposure. Third, antegrade cerebral perfusion can be established if the patient has a more proximal extension of the aortic lesions around LCCA or innominate artery, which is difficult if only the left thoracotomy approach is used.

The L-incision approach is limited in cases with both intracardiac and aortic arch lesions or re-do cases, in which median sternotomy with or without the T-incision or pleural window technique [5] is recommended instead.

## CONCLUSION

A 16-year-old boy with a distal aortic arch aneurysm associated with pseudocoarctation underwent successful distal arch replacement using the L-incision approach. We established total body perfusion without cardiac and circulatory arrest during surgery. The L-incision approach can be a better alternative during aortic arch surgery in adolescents with pseudocoarctation.

**Conflict of interest:** none declared.

## Data Availability Statement

The data underlying this article are available in the article and in its online supplementary material.

## Reviewer information

Interactive CardioVascular and Thoracic Surgery thanks the anonymous reviewer(s) for their contribution to the peer review process of this article.
